# Biodegradation of Deoxynivalenol by *Nocardioides* sp. ZHH-013: 3-*keto*-Deoxynivalenol and 3-*epi*-Deoxynivalenol as Intermediate Products

**DOI:** 10.3389/fmicb.2021.658421

**Published:** 2021-07-19

**Authors:** Honghai Zhang, Heng Zhang, Xing Qin, Xiaolu Wang, Yuan Wang, Yao Bin, Xiangming Xie, Fei Zheng, Huiying Luo

**Affiliations:** ^1^College of Biological Sciences and Biotechnology, Beijing Forestry University, Beijing, China; ^2^Institute of Animal Science, Chinese Academy of Agricultural Sciences, Beijing, China

**Keywords:** deoxynivalenol, 3-*epi*-DON, 3-*keto*-DON, *Nocardioides*, biodegradation

## Abstract

Deoxynivalenol (DON) is one of the most devastating and notorious contaminants in food and animal feed worldwide. A novel DON-degrading strain, *Nocardioides* sp. ZHH-013, which exhibited complete mineralization of DON, was isolated from soil samples. The intermediate products of DON generated by this strain were identified by high-performance liquid chromatography and ultra-performance liquid chromatography tandem mass spectrometry analyses. It was shown that, on an experimental level, 3-*keto*-DON was a necessary intermediate product during the conversion from DON to 3-*epi*-DON. Furthermore, the ZHH-013 strain could also utilize 3-*epi*-DON. This DON degradation pathway is a safety concern for food and feed. The mechanism of DON and 3-*epi*-DON elimination will be further studied, so that new enzymes for DON degradation can be identified.

## Introduction

Deoxynivalenol (vomitoxin, DON) is one of the most important toxic secondary metabolites of mold. Wheat and corn are the main sources of DON in food and animal feed because they are highly important raw food materials that are susceptible to *Fusarium* infection (Xu and Nicholson, 2009). Issues with planting, processing, and warehouse management can cause DON pollution and reduce production yields (Bai and Shaner, 2004). DON has been found to be enriched in water from contaminated cereals during wet processing, in rainwater in the field, and in polluted water from animal farms (Wettstein and Bucheli, 2010; Gracia-Lor et al., 2020; Eagles et al., 2021).

In addition, DON derivatives are important sources of DON for animals and humans. Wheat cultivars and toxigenic *Fusarium* strains can convert DON into masked mycotoxins, such as 3-acetyl DON (3-ADON), 15-acetyl DON (15-ADON), DON-3-glucoside, DON-15-glucoside, DON-sulfate, and DON-glutathione (Miller and Arnison, 1986; Hassan et al., 2016; Jurisic et al., 2019). These masked mycotoxins can then be broken down into DON by gut microbes. Overexposure to DON can lead to intense vomiting in humans and animals, as well as damage to the immune and reproductive systems and disruption of developmental processes (Gunther et al., 2017; Lucke et al., 2017; Springler et al., 2017; Juan-García et al., 2019).

Among the solutions to DON pollution, disease-resistant plants and fungal biocontrol agents are more promising than antifungal agricultural chemicals. Lower disease incidence greatly reduces DON pollution during planting and raw material storage. In addition, it is necessary to develop efficient and environmentally friendly detoxification technologies for processing, waste treatment and ecological restoration.

Heat (Bretz et al., 2006) and ozone (Li et al., 2019) are widely studied physical and chemical DON elimination methods that are considered safe and environmentally friendly and achieve efficient degradation; however, the resulting degradation products are unstable and difficult to analyze because of their complexity, and these methods may reduce the nutritional quality of raw food materials (Santos Alexandre et al., 2018). Therefore, non-thermal methods, such as intense pulsed light (Chen et al., 2019), plasma-activated water (Chen et al., 2019), carbon nitride nested tubes (Bai et al., 2019), and the upconversion nanoparticle @TiO_2_ (Zhou et al., 2020), are of interest because they are environmentally friendly, efficient, and/or economical. These methods are good for processing foods with smooth surfaces or free DON in solvent, and may be useful in the sorting and pretreatment of raw food materials; however, their use to influence animal digestion processes, with the aim of eliminating unreleased or masked DON in food, is less feasible.

Compared with the methods mentioned above, DON biodegradation technologies are gentle, effective, and easy to scale up. Among them, microorganisms or their metabolic detoxification systems that exhibit DON degradation abilities are receiving the most attention.

Massive screening techniques using diverse methods have been conducted to identify DON-degrading microbes, several of which have high DON conversion ability. Over the last three decades, DON-degrading strains have been identified among genera including *Eubacterium* (Fuchs et al., 2002), *Eggerthella* (Gao et al., 2018), *Desulfitobacterium* (He et al., 2020), *Devosia* (He et al., 2016; Wang et al., 2017, 2019), *Sphingomonas* (Ito et al., 2013; He et al., 2017), *Nocardioides* (Ikunaga et al., 2011), *Marmoricola* (Ito et al., 2012), *Bacillus* (Li et al., 2011), *Pelagibacterium* (Zhang et al., 2020), and *Aspergillus* (He et al., 2008; Jaqueline and Eliana, 2010; Jaqueline et al., 2011; Yang et al., 2017).

Extensive epidemiological investigations have shown that many animals are insensitive to DON; however, monogastric animals, especially swine, are sensitive to DON. Rumen and gut microbes are considered to be effective at DON detoxification. Nevertheless, only a few DON-degrading strains have been isolated from rumens or chicken intestines, and they could only transform DON to de-epoxy DON (de-DON or DOM-1) under anaerobic conditions. *Eubacterium* sp. BBSH 797 was the first anaerobic DON-degrading strain isolated from the rumen fluid of a cannulated cow (He et al., 1992; Fuchs et al., 2002). When swine ate feed containing this strain, the negative effect of DON on pig intestines was controlled (Grenier et al., 2013). *Eggerthella* sp. DII-9, isolated from chicken intestines, can similarly transform DON (Gao et al., 2018). In the past, it was thought that the conversion of DON to DOM-1 could only be completed under anaerobic conditions; however, recent studies have shown that *Desulfitobacterium* sp. PGC-3-9 can transform DON under both anaerobic and aerobic conditions (He et al., 2020). The genus *Devosia* has been widely studied, and several species in this genus can detoxify DON. The DON transformation mechanism of *Devosia* sp. 17-2-E-8 is relatively well understood and involves two enzymes from the DON epimerization (Dep) pathway. DON is first converted to 3-*keto*-DON by DepA (Carere et al., 2018a), and then 3-*keto*-DON is transformed to 3-*epi*-DON by DepB (Carere et al., 2018b). *Sphingomonas* sp. KMS1 and S3-4 can utilize DON as a sole carbon source; the former strain could transform DON to 16-hydroxy-DON using a special cytochrome P450 system (Ito et al., 2013), while the latter strain could only convert DON to 3-*keto*-DON (3-*oxo*-DON) and 3-*epi*-DON (He et al., 2017). Recently, two novel strains, from the LZ-N1 consortium, namely, *Pseudomonas* sp. Y1 and *Lysobacter* sp. S1, were shown to exhibit sustained ability to transform DON into the metabolite 3-*epi*-DON, with no degradation products detected after 72 h (Zhai et al., 2019).

In this study, a new DON-degrading strain, designated *Nocardioides* sp. ZHH-013, was isolated from soil associated with a high incidence of root rot. We examined the DON-degrading activity of the species and assessed the detected intermediate products, 3-*keto*-DON and 3-*epi*-DON.

## Materials and Methods

### Soil Samples, Chemicals, and Media

Soil samples (*n* = 1360) were collected from wheat, corn, or forest fields at Jiangsu, Anhui, Hebei, Beijing, Liaoning, Heilongjiang, Nei Mongol, Yunnan, Gansu, and Xinjiang in China, to isolate DON-degrading microbes.

Deoxynivalenol (32943-5MG, Sigma-Aldrich, United States; 16.87 mM in water) was stored at −20°C.

Mineral salt medium (MM) (Ikunaga et al., 2011) was used for enrichment and DON degradation assays. R2A (Difco, United States), Luria-Bertani (LB) broth (Oxoid, United Kingdom), and tryptic soy broth (TSB; Oxoid) were used to culture the DON-degrading strains isolated from soil samples.

### Screening, Enrichment, and Isolation of DON-Degrading Strains

Each soil sample (0.05–0.10 g) was suspended in 0.7 ml of MM with 168.74 μM DON and incubated at 30°C and 220 rpm for 21 days. Thereafter, 20-μl culture aliquots were added to 0.5 ml of MM containing 168.74 μM DON, followed by 21 days of incubation under the same conditions. This process was repeated at least three times. Samples were taken at each step, mixed with an equal volume of sterile 50% glycerol, and stored at 80°C. The concentration of DON in samples was then assessed by high-performance liquid chromatography (HPLC), as described below.

After a series of dilutions, final enriched cultures were spread onto R2A, LB, or TSB agar plates and incubated at 30°C for 14 days. Various colonies were selected and incubated in MM containing 168.74 μM DON at 30°C for 14 days. Thereafter, concentrations of DON were detected by HPLC. Pure cultures were mixed with an equal volume of sterile 50% glycerol and stored at −80°C. All screening and incubation processes were carried out under aerobic conditions.

### Phylogenetic Analysis of *Nocardioides* sp. ZHH-013

The partial 16S rRNA gene sequence of the ZHH-013 strain was amplified by PCR using primers 27f and 1492r (Zhang et al., 2017). The resulting amplicon was an approximately 1381-bp 16S rDNA sequence (GenBank Accession No. MW493343). Similar sequences were identified using BLAST (Johnson et al., 2008) and 16S-based ID apps in the EzBioCloud server (Yoon et al., 2017). Neighbor-joining phylogenetic trees were constructed using MEGA X software (Kumar et al., 2018). Alignment was conducted using the ClustalW program (Higgins et al., 1994). Bootstrap analysis was performed with 1,000 replications.

### DON Degradation Assay

To obtain an initial culture, a single ZHH-013 colony was inoculated in 700 μl of MMD and grown under aerobic conditions at 30°C for 8–12 days.

To assess DON degradation by a high-density culture of the ZHH-013 strain, 3 ml of TSB medium was mixed with the initial culture (1%) and grown at 30°C, with agitation at 220 rpm for 7 days. Next, culture densities were adjusted to an OD_600_ of 0.4–0.6, inoculated in 100 ml of TSB, and incubated at 30°C and 220 rpm for 5 days.

Next, 50 ml of fermentation broth was transferred to a 50-ml centrifuge tube and centrifuged at 14,400 × *g* for 3 min at 4°C. The supernatant was collected and filtered through a 0.22-μm filter (Millipore, Cork, Ireland) to generate a cell-free supernatant. Additionally, the cell pellets were washed three times with sterilized water and resuspended in 5 ml of MM. Next, DON was added to the samples at the final concentrations of 0.169, 1.687, 3.375, and 33.747 mM. All samples were incubated at 30°C with 220 rpm for 10 and 24 h.

In addition to the fermentation broth supernatant and the cell pellets, we also assessed lysed cells. Cells were lysed using a sonic disrupter, and lysates centrifuged at 20,700 × *g* (4°C, 20 min) to obtain supernatants. Aliquots were left untreated or filtered using 0.22-μm filters. DON (168.74 μM) was added to the samples and incubated at 30°C and 220 rpm for 10 and 24 h.

All samples were stored at −80°C before analysis by HPLC or ultra-performance liquid chromatography tandem mass spectrometry (UPLC-MS/MS), as described below.

### Preparation and Purification of 3-*keto*-DON and 3-*epi*-DON

To prepare 3-*keto*-DON as the standard control (Carere et al., 2018a), the synthesis gene of *dep*A from *Devosia* sp. 17-2-E-8 was purchased from GENEWIZ (Su Zhou, China). The gene was cloned into pET-28a (+) and then transformed into *Escherichia coli* BL21(DE3). After ensuring inducible expression and purification by Ni-nitrilotriacetic acid chromatography, DepA was mixed with 0.1 mM KH_2_PO_4_, 0.1 mM NaOH, 1 mM Ca^2+^, and 100 μM pyrroloquinoline quinone, and then incubated at 30°C for 20 h. Thereafter, a triple volume of methanol was added and samples were left at −20°C for 20 min. After centrifugation (4°C, 20 min), the supernatant was collected. The solvent was removed using vacuum freeze-drying equipment (Martin Christ, Osterode am Harz, Germany), and 3-*keto*-DON was dissolved in 100 μl of methanol. The 3-*keto*-DON standard was stored at −80°C before HPLC or UPLC-MS/MS analysis.

Preparation of 3-*keto*-DON produced by ZHH-013 strain referred to the analysis method as described above. Preparation of 3-*epi*-DON produced by ZHH-013 strain refers to the method described by Ikunaga et al. (2011) with modification. Wet *Nocardioides* sp. ZHH-013 cells were resuspended in 1 ml of MM (OD_600_ = 1.0) containing 10 mg of DON, and incubated for 41 h at 30°C. After centrifugation at 20,700 × *g* (4°C, 20 min), the supernatant was collected and filtered using a 0.22-μm filter. The solvent was drawn off completely using vacuum freeze-drying equipment (Martin Christ) and the sample was resuspended in 100 μl of methanol. Analytical HPLC (described in the next section) was used to prepare 3-*epi*-DON and 3-*keto*-DON. All elution fractions of 3-*epi*-DON and 3-*keto*-DON were mixed, respectively. After sample enrichment by vacuum freeze-drying (Martin Christ) and resuspension in 100 μl of methanol, all degradation products were stored at −80°C before HPLC or UPLC-MS/MS analysis.

### HPLC Analysis of DON, 3-*epi*-DON, and 3-*keto*-DON

Equal volumes of samples were mixed well with methanol stored at −20°C. Before HPLC analysis, samples were filtered using 0.22-μm filters.

The HPLC system (Shimadzu, Kyoto, Japan) consisted of an LC-20AT pump and an SPD-20A UV/VIS detector. A reverse phase column (Agilent TC-C18, 4.6 mm × 250 mm, 80 Å, 5 μm) was used. To detect DON and its derivatives, the mobile phase comprised methanol and water (15:85, v/v) at a flow rate of 1.0 ml/min. To detect 3-*keto*-DON, the mobile phase comprised methanol and water (1:2, v/v). UV/VIS detection was performed at a wavelength of 220 nm. The column was heated to 40°C.

### UPLC-MS/MS Analysis of DON, 3-*epi*-DON, and 3-*keto*-DON

UPLC-MS/MS analysis was performed using a Nexera UHPLC LC-30A (Shimadzu) UPLC system coupled with TripleTOF5600 (AB Sciex, United States). For C18 separation, mobile phase A was acetonitrile, and mobile phase B was 0.5% formic acid in water. The column was an HSS T3 column (150 mm × 3 mm, 1.8 μm; Waters) operated at 40°C. The flow rate was 300 μl/min and the injection volume was 1 μl. Gradient conditions were as follows: 0–10 min, A: 0 to 50%, B: 100 to 50%; 10–13 min, A: 50 to 95%, B: 50 to 5%; 13–14 min, A: 95 to 0%, B: 5 to 100%; 14–15 min, A: 0 to 0%, B: 100 to 100%.

The mass spectrometer was operated in positive ion mode with ion spray voltage floating, 5500 V; ion source gas 1 50 psi; ion source gas 2 50 psi; and curtain gas at 25 psi and source temperature, 500°C. Sample analysis was performed by information-dependent acquisition, with a 200-ms time-of-flight (TOF)-MS scan from 100 to 1,500 Da, followed by an MS/MS scan in high-sensitivity mode from 50 to 1,500 Da of the top 20 precursor ions from the TOF-MS scan.

## Results and Discussion

### Screening, Identification, and Characterization of *Nocardioides* sp. ZHH-013

Microbes are the main decomposers in nature and soil is the richest source of strains with degradation activity. Over the last three decades, especially the past 3 years, several strains that can efficiently transform DON have been reported. *Nocardioides* sp. WSN05-2, isolated from wheat field soil, was the first *Nocardioides* strain demonstrated to degrade DON as a carbon source (Ikunaga et al., 2011). It completely converted DON after 10 days of incubation and no signal of a typical trichothecene skeleton was detected by nuclear magnetic resonance (NMR) analysis (Ikunaga et al., 2011); however, no more public information has been released about the DON degradation function of this genus, despite several researchers continuing to screen for DON degrading microorganisms.

Enrichment culture of 1,360 soil samples was conducted; after three rounds of screenings, 24 samples with DON-degrading activity were isolated. Among them, 13 samples exhibited DON degradation characteristics similar to those reported for *Devosia*. The remaining 11 samples could completely transform DON. After 10 sub-inoculations, Culture Sample No. 13 showed stable and efficient DON-degrading activity. Microbial diversity analysis of Sample No. 13 showed that *Nocardioides* (45.20%) and *Paracoccus* (27.25%) were the main components at the genus level (unpublished data).

**FIGURE 1:**
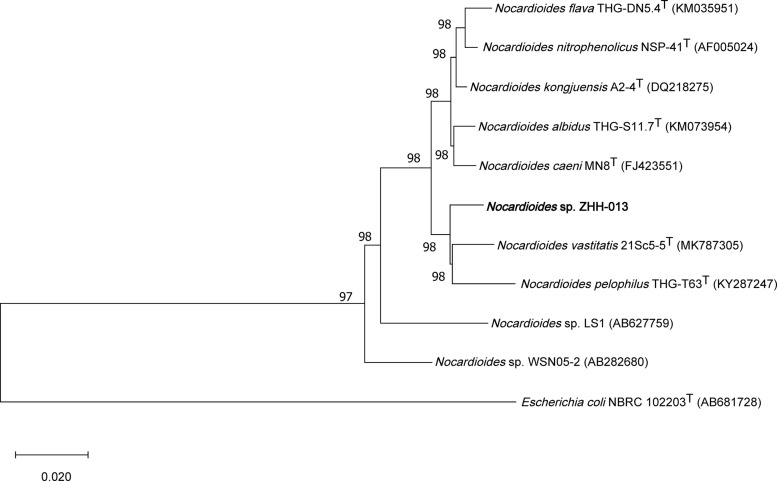
Phylogenetic tree, including *Nocardioides* sp. ZHH-013, based on neighbor-joining analysis of 16S rDNA sequences.

An enriched culture of Sample No. 13 was plated on various media (LB, TSA, R2A, or MM) using the dilution method. Consequently, the ZHH-013 strain was isolated and a 1,381-bp sequence of its 16S rDNA was used for phylogenetic analysis. Sequences with the highest similarities to the ZHH-013 strain were as follows: *Nocardioides vastitatis* 21Sc5-5^T^ (97.68%), *Nocardioides kongjuensis* A2-4^T^ (97.39%) *Nocardioides nitrophenolicus* NSP 41^T^ (97.32%), *Nocardioides caeni* MN8^T^ (97.25%), *Nocardioides albidus* THG-S11.7^T^ (97.25%), *Nocardioides flava* THG-DN5.4^T^ (97.18%), and *Nocardioides pelophilus* THG-T63^T^ (97.18%). A phylogenetic tree, constructed using the neighbor-joining method, showed that the ZHH-013 strain belongs to the genus *Nocardioides* (Figure 1). The 16S rRNA similarity was lower than the recommended classification threshold of 98.65% (Kim et al., 2014); hence, we inferred that the ZHH-013 strain may be a new *Nocardioides* species. The sequence similarity between ZHH-013 and other DON-degrading *Nocardioides* strains (Sato et al., 2012) was less than or equal to 92.27%.

**FIGURE 2:**
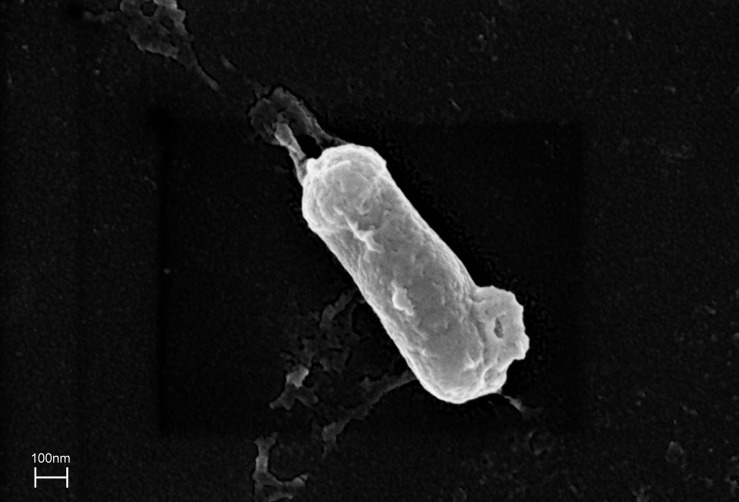
Morphological characteristics of *Nocardioides* sp. ZHH-013 cell (×50,000).

The ZHH-013 strain is a Gram-positive, aerobic, rod-shaped, non-spore-forming bacterium that is 0.28–0.29 × 0.86–0.87 μm in size (Figure 2). Colonies were white and translucent on R2A plates, and yellowish white on LB or TSA plates. The ZHH-013 strain could use DON as a sole carbon source, whereas related species (*N. vastitatis* 21Sc5-5^*T*^, *N. kongjuensis* A2-4^*T*^, *N. nitrophenolicus* NSP 41^T^, *N. caeni* MN8^T^, and *N. albidus* THG-S11.7^T^) could not.

**FIGURE 3:**
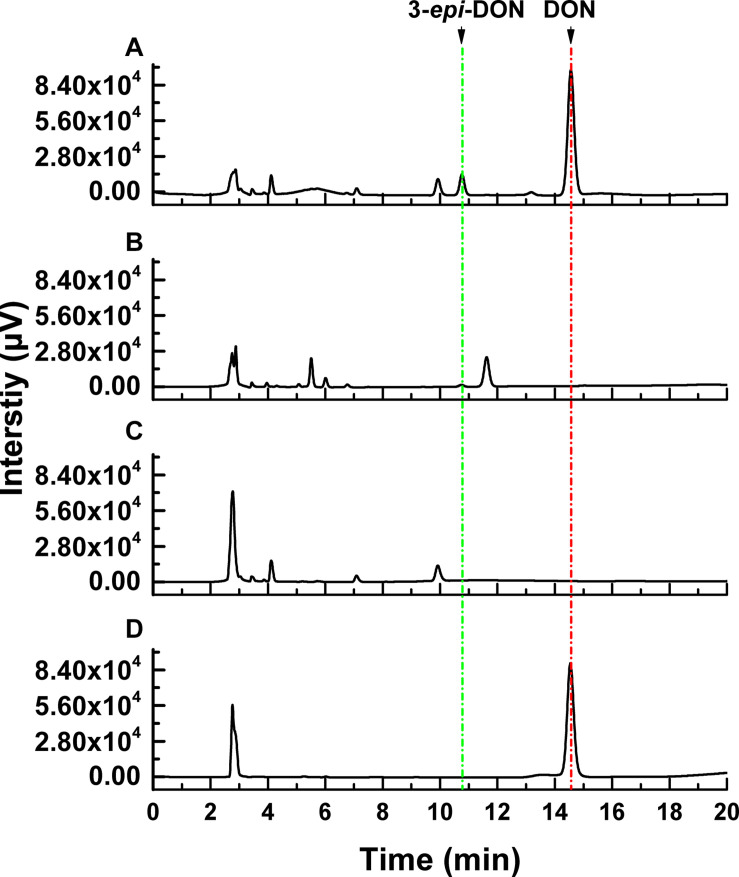
High-performance liquid chromatography analysis of DON degradation products after incubation of DON with **(A)** filtered intracellular supernatant treated, **(B)** intracellular supernatant, and **(C)** untreated *Nocardioides* sp. ZHH-013 cells. **(D)** The 168.74 μM DON standard was used as control.

The DON degradation rate was directly proportional to the amount of ZHH-013 strain. After 48 h of incubation, the DON degradation rate was 80% at an OD_600_ of 0.4 and only 40% after 10-fold dilution. After 10 h of co-incubation with DON, DON was not degraded by ZHH-013 fermentation broth supernatant or filtered or moist heat sterilized (121°C, 20 min) by supernatant of ZHH-013 cell lysate (Figure 3). Thus, the complete degradation capacity of DON may rely on live cells.

### Characterization of DON Biodegradation by *Nocardioides* sp. ZHH-013

Researchers have confirmed DON detoxification mechanisms in several organisms, including animals and plants, and most of these processes rely on microorganisms (Fuchs et al., 2002; Ito et al., 2012; Gao et al., 2018). The five main degradation pathways detected in microorganisms are as follows: (1) DON to DOM-1; (2) DON to 3-ADON or 15-ADON; (3) DON to 3-*keto* DON; (4) DON to 16-DON; and (5) two-step transformation: DON to 3-*keto*-DON and 3-*keto*-DON to 3-*epi*-DON. Some other strains, such as *Aspergillus niger* As-D.1 (Yang et al., 2017), exhibit different mechanisms; however, the degradation products have not been clearly explained based on detailed data. Moreover, the ZHH-013 strain exhibited a new degradation mechanism that differed from the five mechanisms mentioned above.

**FIGURE 4:**
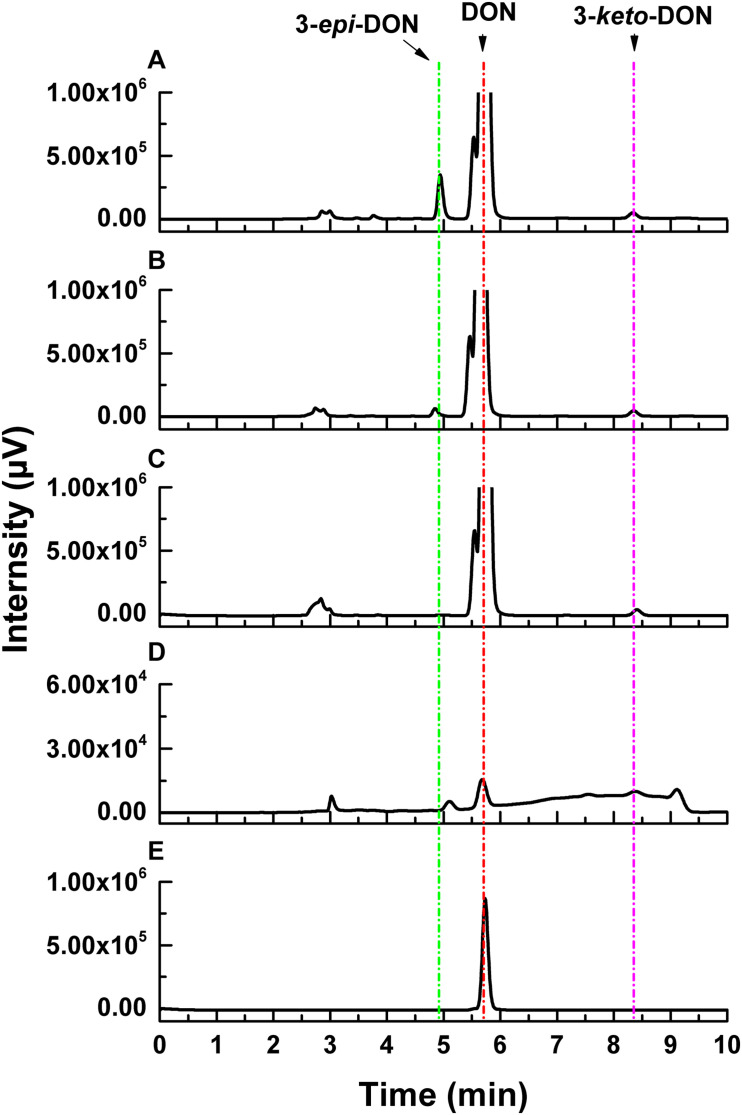
High-performance liquid chromatography analysis of DON degradation by *Nocardioides* sp. ZHH-013 after **(A)** 8 h, **(B)** 4 h, **(C)** 0.5 h, and of **(D)** 3-*keto*-DON produced by DepA. **(E)** The 3.37 mM DON standard.

**FIGURE 5:**
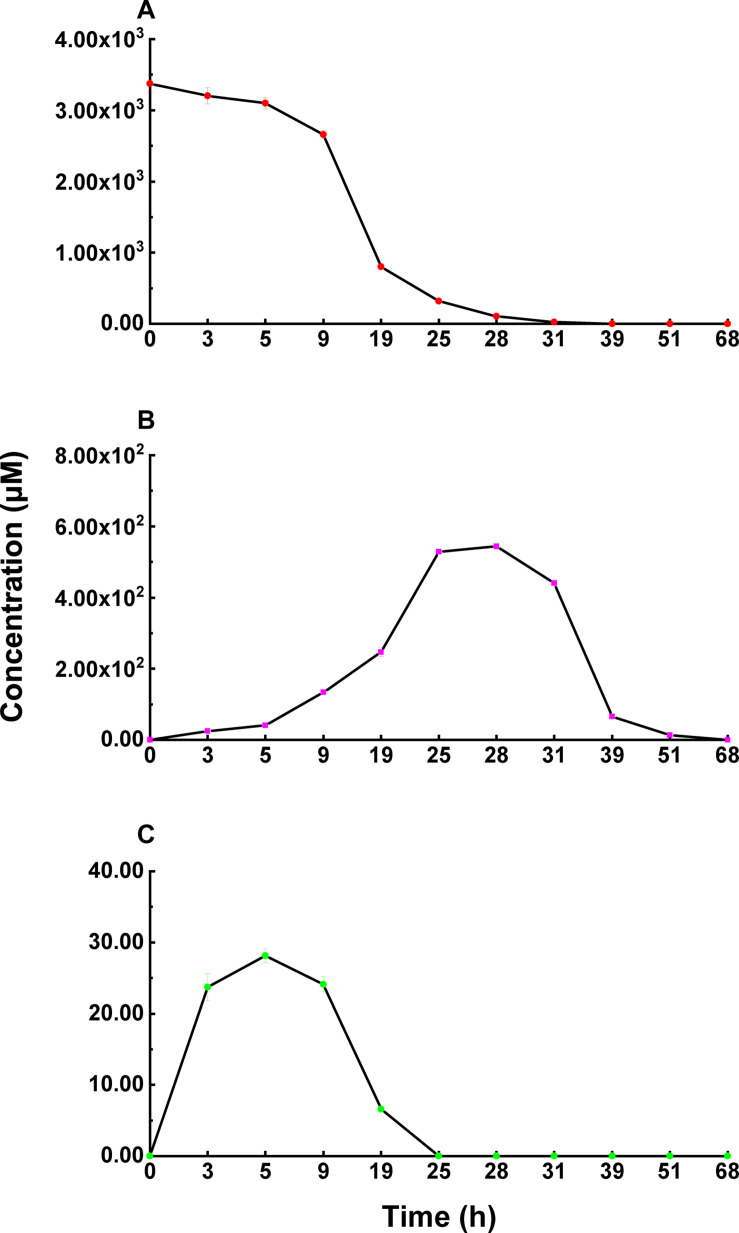
The concentration changes of 3-*epi*-DON **(B)** and 3-*keto*-DON **(C)** in the process of DON **(A)** degradation by *Nocardioides* sp. ZHH-013.

Deoxynivalenol degradation products generated by ZHH-013 were detected by HPLC and UPLC-MS/MS. 3-*epi*-DON appeared as an early DON degradation product (Figures 4, 5). Based on MS and MS/MS data indicating a compound with a retention time (RT) of 3.09 min (Supplementary Figure 2A), the predominant ion at *m/z* 297.13 was consistent with a molecular ion of [DON + H]^+^ (Supplementary Figures 2B,C), which was similar to that of DON (Supplementary Figures 1B,C). 3-*epi*-DON has previously been identified as a DON degradation product, based on MS and NMR data (Ikunaga et al., 2011; He et al., 2015b). After continued incubation, the peak for 3-*epi*-DON surprisingly disappeared (Figure 5B and Supplementary Figure 5) and we concluded that ZHH-013 could also utilize 3-*epi*-DON. These results resemble the findings reported for the strain, WSN05-2 (Ikunaga et al., 2011), which can utilize 3-*epi*-DON as a sole carbon source.

**FIGURE 6:**
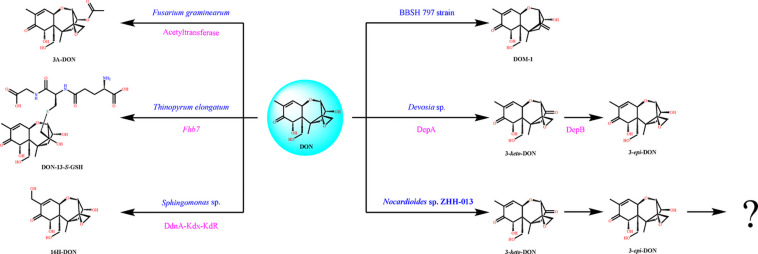
Typical DON degradation pathways of different microorganisms including *Nocardioides* sp. ZHH-013. The blue characters denote microorganism name, and the rose red characters denote identified gene or enzyme.

Karlovsky speculated that *Nocardioides* sp. WSN05-2 may provide a high ratio of oxidation and reduction rates, leading to low accumulation of the intermediate product (3-*keto*-DON) (Karlovsky, 2011); however, no experimental evidence was published. Using the ZHH-013 strain, we observed very little accumulation of DON degradation product, 3-*keto*-DON, unlike reports of experiments using DepA obtained from *Devosia* sp. 17-2-E-8 (Figures 4, 5C); however, after further analysis, a new peak (RT = 4.06 min) was identified as 3-*keto*-DON. MS and MS/MS data from that compound revealed a predominant ion at *m/z* 293.10, consistent with the molecular ion [DON–2–H]^–^ (Supplementary Figures 4B,C), similar to that of 3-*keto*-DON prepared from DON using DepA (Supplementary Figures 3B,C). The difference of 2 atomic mass units between the ions at *m/z* 293.10 [DON–2H–H]^–^ and *m/z* 297.13 [DON + H]^+^ indicates that two protons were absent. 3-*keto*-DON disappeared when DON was depleted, and no 3-*keto*-DON accumulation was found when 3-*epi*-DON was degraded. Therefore, we presumed that DON must have been converted to 3-*keto*-DON before then being converted to 3-*epi*-DON (Figure 6) and that the efficient conversion ability of *Nocardioides* sp. ZHH-013 may have led to low accumulation of 3-*keto*-DON.

The toxicity of DON degradation products is a core question for strain or enzyme applications. The toxicity of DON has been fully studied, while *in vitro* and *vivo* toxicological data on its derivatives remain very scarce. The 12,13-epoxide ring, hydroxyl group at C-15, hydroxyl group at C-3, and double bond between C-9 and C-10 are crucial DON toxic groups (McCormick et al., 2011). DON inhibits nucleic acid synthesis by binding to peptidyl transferase in the 60S ribosomal subunit, inducing ribotoxic stress, and activating MAPKinases (Maresca, 2013; Garreau de Loubresse et al., 2014). Unlike DON, 3-*epi*-DON does not activate MAPKinases (Pierron et al., 2016). The toxicity of DON derivatives created by microorganisms has been tested using BrdU bioassays (Widestrand et al., 1999; Sundstøl et al., 2004). The toxicity of 3-*epi*-DON is substantially lower than that of DON and its derivatives, while excessive exposure to 3-*epi*-DON still caused organ lesions in mice (He et al., 2015a). Moreover, exposure to 3-*epi*-DON caused slight changes in intestinal explants (Pierron et al., 2016); however, 3-*epi*-DON showed almost no hepatotoxicity or immunotoxicity in *in vivo* pig experiments (Bracarense et al., 2020). Therefore, 3-*epi*-DON may represent a low-toxicity, safe DON degradation product in mice and pigs. The toxicity of 3-*epi*-DON is potentially due to the 12,13-epoxide ring and the double bond between C-9 and C-10 (Wei and McLaughlin, 1974). Therefore, elimination of 3-*epi*-DON may be necessary. *Nocardioides* sp. ZHH-013 metabolizes 3-*epi*-DON, and we speculate that it may induce the oxygen heterocyclic form of 3-*epi*-DON to undergo a ring-opening reaction in one or more steps, and that the toxicity of DON can thereby be eliminated.

## Conclusion

Biodegradation is an effective strategy to reduce toxin exposure risks and economic losses. There are five important issues that have been hindering the development of industrial bacteria and enzyme preparations: (1) the lack of strains or enzyme sources, (2) the safety of the transformed products, (3) the lack of compound toxicology data, (4) the safety of the strains, and (5) the industrial application of the strains and enzymes.

In this paper, we report a novel DON-degrading strain, *Nocardioides* sp. ZHH-013, isolated in China. ZHH-013 can degrade DON to 3-*keto*-DON and 3-*epi*-DON, achieving DON mineralization. The DON degradation mechanism of this strain may inspire the development of attenuated agents or bio-detoxifiers. Determining the mechanisms underlying the efficient generation and degradation of 3-*epi*-DON may lead to the discovery of new enzymes. Furthermore, there is potential to improve enzyme activity, expression level, and adaptability *via* enzyme engineering technology, which would help to realize the application of DON-degrading enzymes to eliminate DON pollution in food/feed fields or to restore DON-polluted bodies of water on farms.

## Data Availability Statement

The datasets presented in this study can be found in online repositories. The names of the repository/repositories and accession number(s) can be found below: https://www.ncbi.nlm.nih.gov/genbank/, MW493343.

## Author Contributions

HoZ and HeZ performed the experiments. FZ and YB designed and performed the synergism experiments and analyzed the data. XQ, XW, and XX designed the research and collected soil samples. HoZ, FZ, and HL revised the manuscript. All authors read and approved the final manuscript.

## Conflict of Interest

The authors declare that the research was conducted in the absence of any commercial or financial relationships that could be construed as a potential conflict of interest.

## Abbreviations

15-ADON: 15-acetyl DON
3-*epi*-DON: 3-*epi*-deoxynivalenol
3-*keto*-DON: 3-*keto*-deoxynivalenol
Dep: DON epimerization
DOM-1: de-epoxy DON
DON: deoxynivalenol
HPLC: high-performance liquid chromatography
LB: Luria-Bertani
MM: mineral salt medium
MS: mass spectrometry
MS/MS: tandem mass spectrometry
NMR: nuclear magnetic resonance
RT: retention time
TSB: tryptic soy broth
UPLC: ultra-performance liquid chromatography.
