# Supplementary material for: Biodegradation of Deoxynivalenol by Nocardioides sp. ZHH-013: 3-keto-Deoxynivalenol and 3-epi-Deoxynivalenol as Intermediate Products
Source: Front Microbiol. 2021 Jul 19;12:658421. doi: 10.3389/fmicb.2021.658421 (PMC8326517; doi:10.3389/fmicb.2021.658421)
Supplement: Supplementary file 1 [file Data_Sheet_1.docx]

***Supplementary Material***


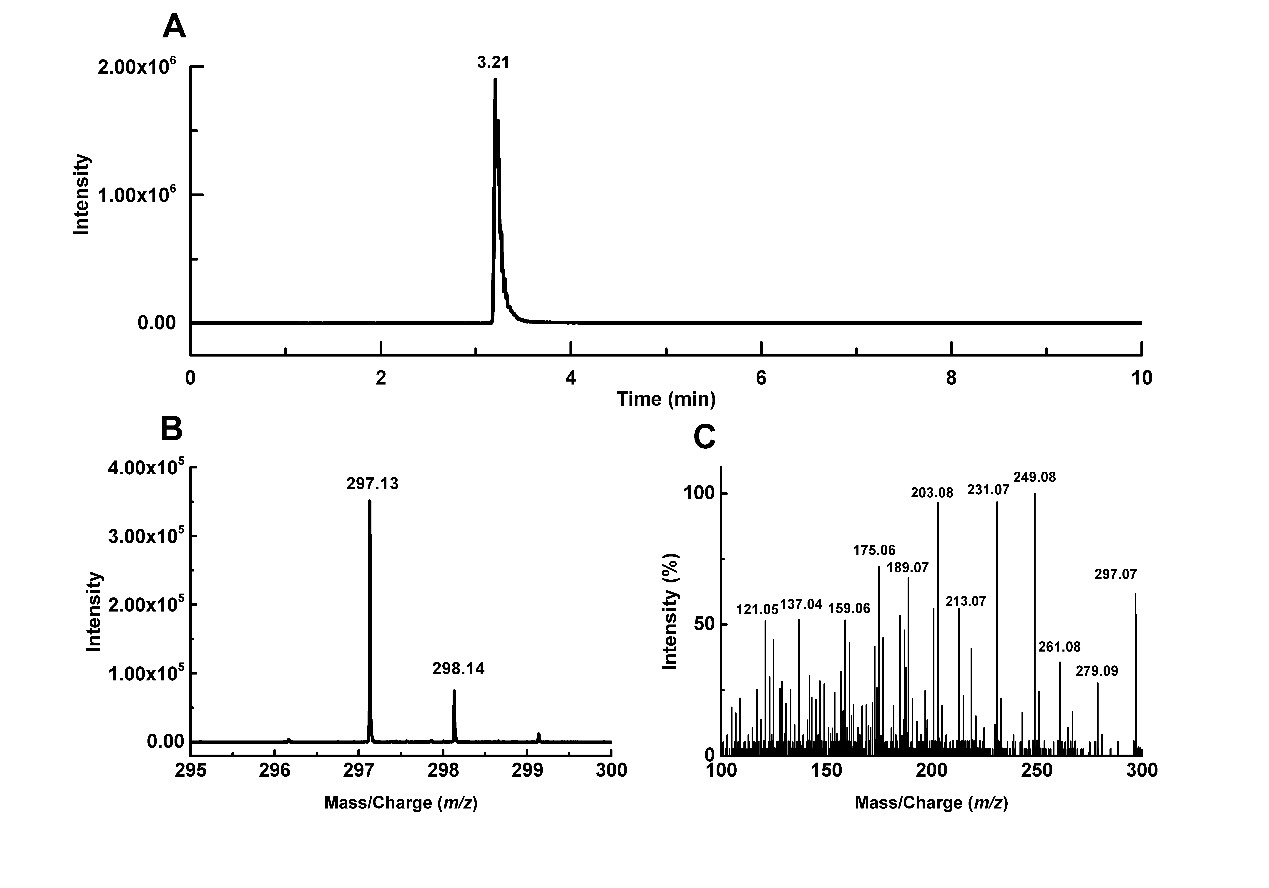


**Supplementary Figure** **1.** Extracted ion chromatogram **(A)**, Mass spectra **(B)** and MS/MS **(C)** of deoxynivalenol analyzed by positive-ion-mode UPLC-ESI-MS/MS.


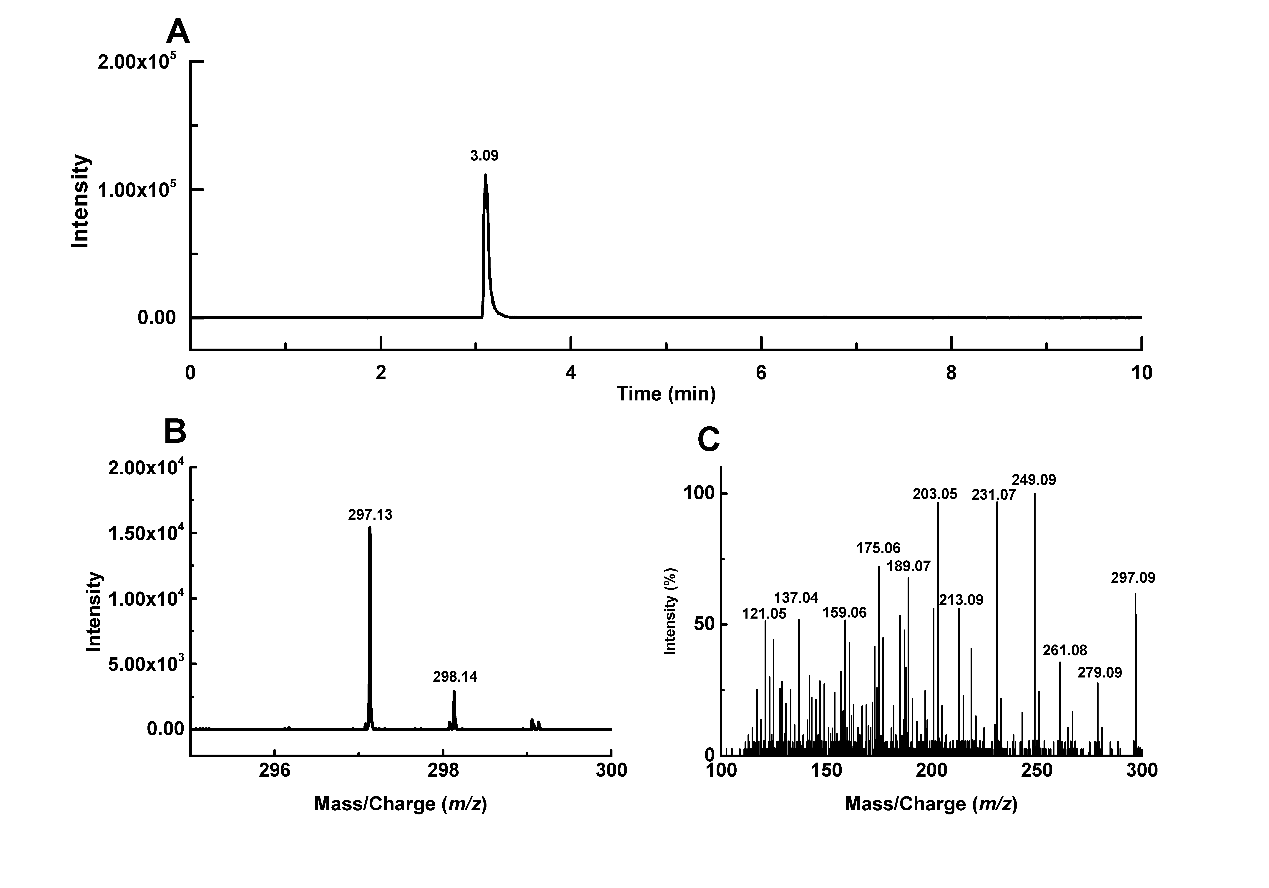


**Supplementary Figure** **2.** Extracted ion chromatogram **(A)**, Mass spectra **(B)** and MS/MS **(C)** of 3-*epi*-deoxynivalenol analyzed by positive-ion-mode UPLC-ESI-MS/MS.


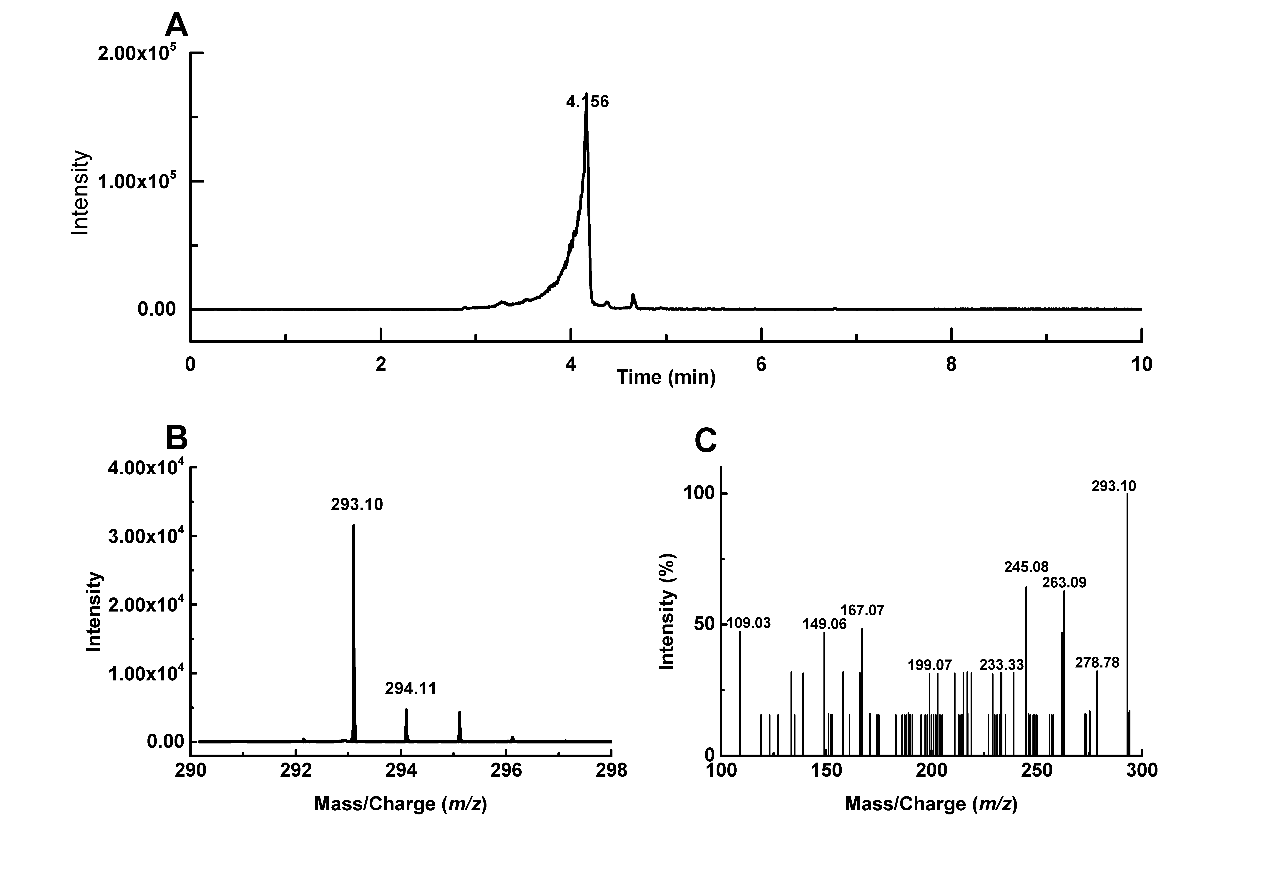


**Supplementary Figure** **3.** Extracted ion chromatogram **(A)**, Mass spectra **(B)** and MS/MS **(C)** of 3-*keto*-deoxynivalenol produced by DepA analyzed by negative-ion-mode UPLC-ESI-MS/MS.


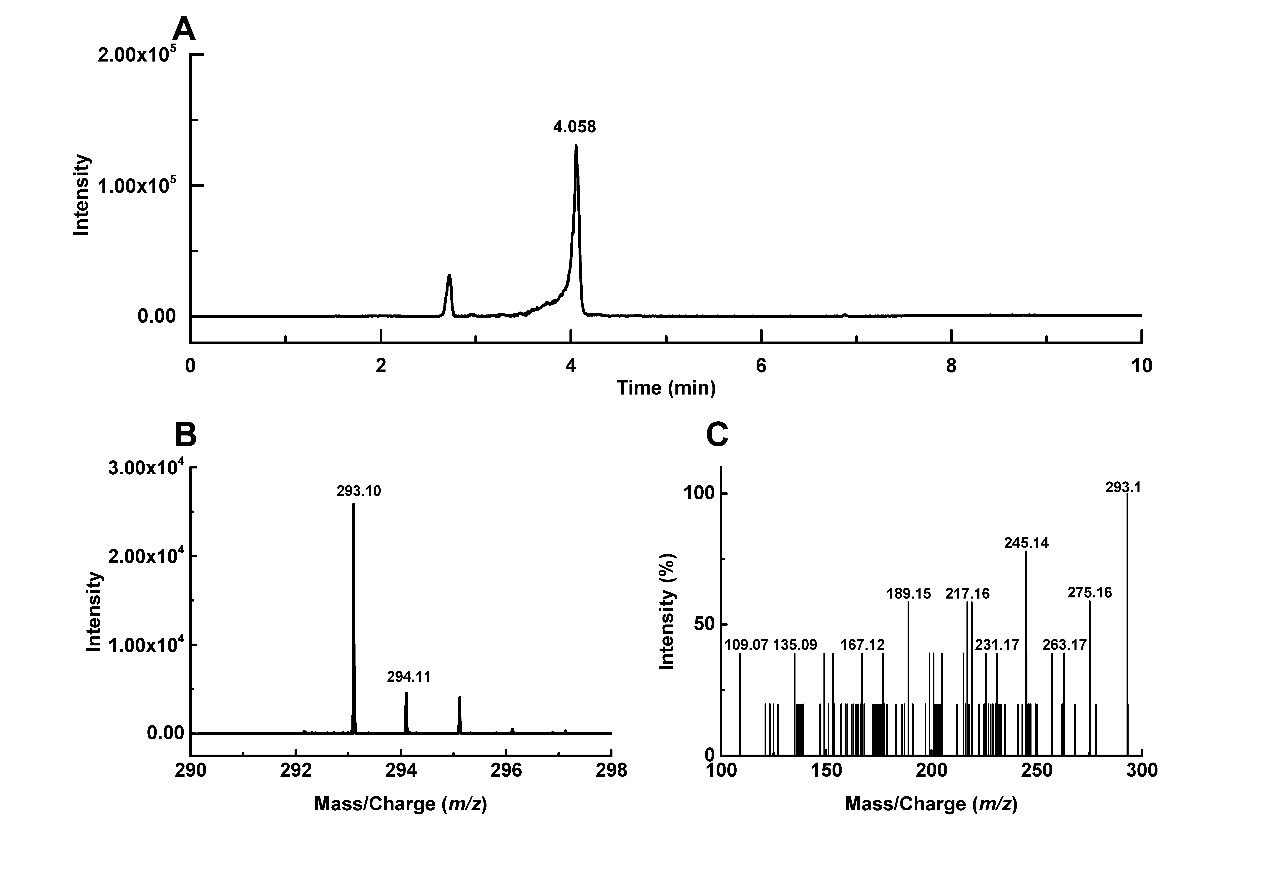


**Supplementary Figure** **4.** Extracted ion chromatogram **(A)**, Mass spectra **(B)** and MS/MS **(C)** of 3-*keto*-deoxynivalenol produced by *Nocardioides* sp. ZHH-013 analyzed by negative-ion-mode UPLC-ESI-MS/MS.


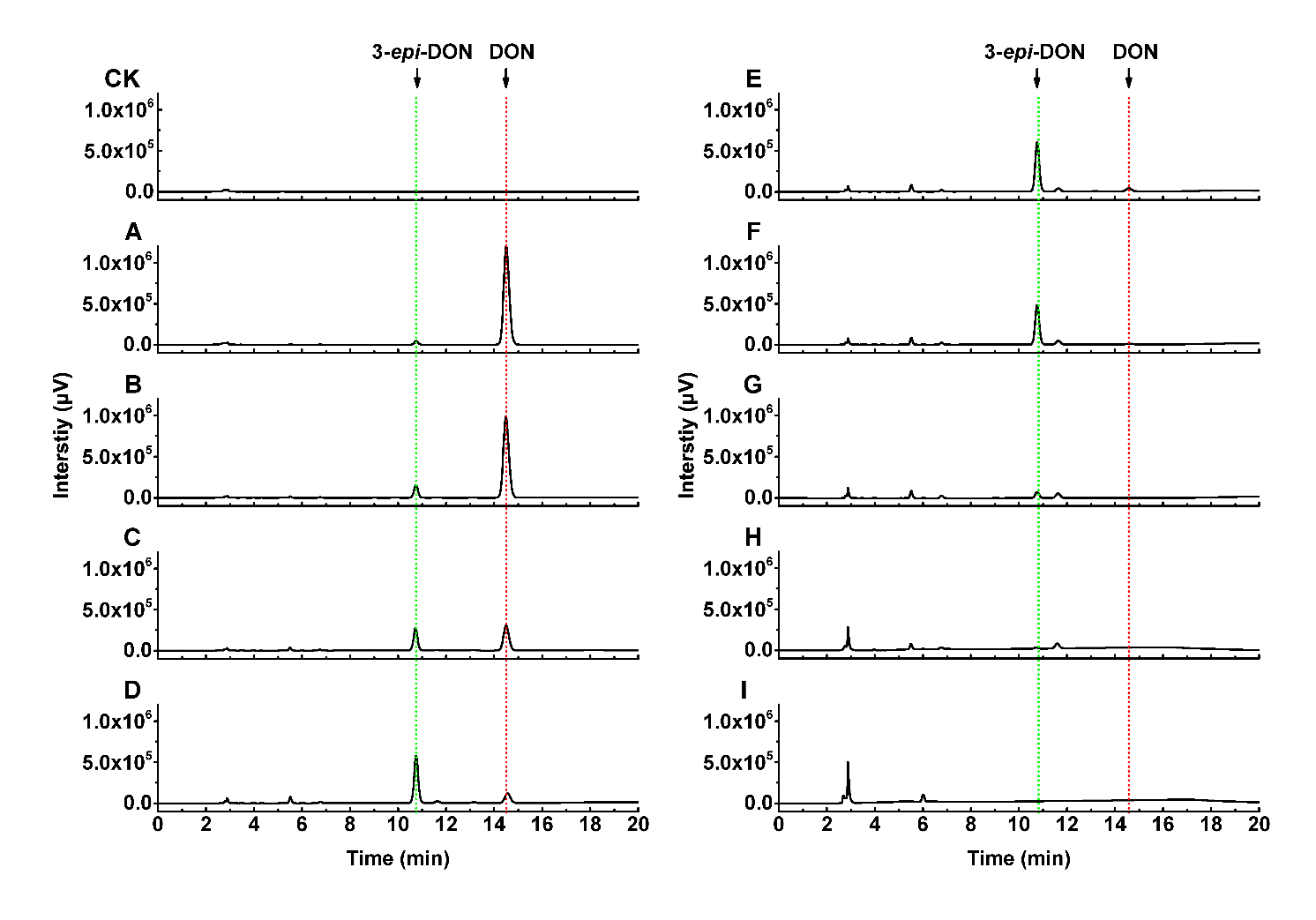


**Supplementary Figure** **5.** High-performance liquid chromatography analysis of DON and its degradation products after incubation with *Nocardioides* sp. ZHH-013 for **(A)** 5, **(B)** 9, **(C)** 19, **(D)** 25, **(E)** 28, **(F)** 31, **(G)** 39, **(H)** 51, and **(I)** 68 h. Samples without DON were used as controls **(CK)**.
